# Influences on the Decision to Euthanize a Compromised Pig

**DOI:** 10.3390/ani14152174

**Published:** 2024-07-26

**Authors:** Julia Stoffregen, Tristan Winkelmann, Bettina Schneider, Michel Fehrmann, Kathrin Gerdes, Moana Miller, Jennifer Reinmold, Isabel Hennig-Pauka, Nicole Kemper, Christin Kleinsorgen, Karl-Heinz Tölle, Lothar Kreienbrock, Michael Wendt, Elisabeth grosse Beilage

**Affiliations:** 1 Institute of Biometry, Epidemiology and Information Processing (IBEI), WHO-CC for Health at the Human-Animal-Environment Interface, University of Veterinary Medicine, Foundation, Hannover, Bünteweg 2, 30559 Hannover, Germany; 2 Field Station for Epidemiology, University of Veterinary Medicine, Foundation, Hannover, Büscheler Str. 9, 49456 Bakum, Germany; 3 Institute for Animal Hygiene, Animal Welfare and Farm Animal Behavior, University of Veterinary Medicine, Foundation, Hannover, Bischofsholer Damm 15, 30173 Hannover, Germany; 4 Centre for E-Learning, Didactics, Educational Research [ZELDA], University of Veterinary Medicine, Foundation, Hannover, Bünteweg 2, 30559 Hannover, Germany; 5 ISN-Projekt GmbH, Kirchplatz 2, 49401 Damme, Germany; 6 Clinic for Swine, Small Ruminants, Forensic Medicine and Ambulatory Service, University of Veterinary Medicine, Foundation, Bischofsholer Damm 15, 30173 Hannover, Germany

**Keywords:** challenges, decision-making, pigs, attitudes, justification, clinical reasoning, clinical signs, timely euthanasia

## Abstract

**Simple Summary:**

The decision to euthanize a compromised pig can be difficult for pig farmers and veterinarians. To understand more about the challenges in Germany, an online survey was conducted on the reasons, influences, and attitudes of participants about the timely euthanasia of a compromised pig. The results of the survey showed that a multitude of influences, due to the work context on the farm and economic and personal considerations, shape the decision-making process. The reasons outlined by German participants correspond to those published in similar studies about the topic. However, this study asked for specific influences, including the delay of timely euthanasia. In this regard, the uncertainty and misinterpretation of the chance for healing were outlined as the two most salient factors. Moreover, a lack of valid clinical signs and disagreements about decisions were outlined as major influences. In summation, this study outlines the need to generate further evidence about the development of clinical signs in compromised pigs and to improve clinical reasoning skills to enhance the training and consultation of farmers and veterinarians.

**Abstract:**

The decision to euthanize a compromised pig can be challenging for pig farmers and veterinarians. To understand more about the challenges in Germany, a cross-sectional online survey was conducted. Based on a hybrid design, the responses of 39 veterinarians and 62 pig farmers were analyzed to generate a list of common clinical signs associated with the euthanasia of sows, fatteners, and piglets. Moreover, a set of influences on the farm, due to economic and personal considerations, were found to shape the decision-making process. The two most salient reasons outlined for the delay of timely euthanasia were uncertainty and misinterpretation of the chance for healing. The lack of valid clinical signs or a sound justification was most frequently mentioned as a challenge to the general decision-making process. In summation, this study highlights the need to generate a valid taxonomy for clinical signs that includes their development in a compromised pig over time. Future studies should elaborate on the justification of euthanasia decisions to facilitate the resolution of ethical dilemmas among the involved pig farmers and veterinarians. Lastly, the results suggest that clinical reasoning and consultation skills should be included when decision-making behavior is to be trained.

## 1. Introduction

To secure the well-being of pigs, farmers and veterinarians evaluate the state of individual animals and the herd up to several times a day. While it is possible for an animal to become diseased or injured from time to time, some conditions require decisions about euthanizing the affected pig. The evaluation depends on the condition of the individual pig and serves to avoid unnecessary suffering and pain if healing is impossible or no alternate relief can be provided. Often, the evaluation is challenging since pigs tend to hide their pain or impairment. Moreover, the situation on farms can shape the decision-making process, including the influence of individual attitudes among external factors, such as economic considerations. Since recent studies show that for some pigs, the decision to euthanize is delayed or even omitted, the decision-making process and influences on timely euthanasia have become salient research topics in veterinary science and practice [1,2,3,4].

To improve understanding, one focus of recent studies has been to generate knowledge about typical diseases, disorders, and clinical signs related to the euthanasia of pigs. In this regard, post-mortem examinations [5,6,7], surveys of caretakers and veterinarians [4], and literature reviews [8] have been conducted. These studies have resulted in lists of common diseases in which a gap was found for reported cases of suckling pigs, weaners, and fatteners. Moreover, methods for harmonizing the reporting of both symptoms and etiological diseases remain a research question to be solved [6].

Knowledge about common diseases has been used in the training of caretakers to facilitate the identification of compromised pigs on farms [9]. However, the effect of knowing about common diseases on timely euthanasia is difficult to assess, and it may not help to secure timely decision-making as such [10]. A second focus of recent studies has thus been to elaborate on influences on the decision to euthanize a compromised pig. Categories of influences address the setting and cultural context of the workplace, attitudes toward euthanasia, the level of skills and training [10,11,12,13,14], economic considerations [15,16], and technical concerns [17,18]. Both the list of categories and the nature of the influencing factors need to be further disentangled. More precisely, it is suggested to assess opinions from veterinarians and farmers jointly and to ask about influences that facilitate and challenge the decision-making process in particular [5,8,12].

Associated with the influences on the decision to euthanize a compromised pig is that decisions are rarely made in one instance. Instead, the condition of the pig is re-evaluated several times. In this respect, alternate decisions such as separating the pig from the group, re-evaluating its state, and, more generally, the process of decision-making over time should be surveyed as well. Consequently, the timing of influences on the decision-making process should be surveyed without pre-defining a time frame [4,8,19].

This brief summary of previous publications shows that a lot of work has been carried out to enhance the knowledge about the timely euthanasia of a compromised pig. However, there are still knowledge gaps, and research has been published in rather few cultural contexts [14]. In view of these considerations, an online survey was designed and conducted with pig farmers and veterinarians in Germany.

## 2. Materials and Methods

This survey was conducted from October until December 2022 along with the project CARE PIG. The project aimed to enhance the knowledge about the critical time point for euthanasia with regard to clinical signs and decision-making processes during the evaluation of individual pigs (CARE PIG). Related to the research questions and the assumptions discussed above within the German context, the goals of this survey were as follows:

- To outline what reasons veterinarians and farmers mention for euthanasia of certain pig categories (sow, piglet, weaner, and fattener);
- To generate a catalog of the most-occurring clinical signs or diseases that require euthanasia of a pig;
- To validate and complement factors that influence the decision-making process to euthanize a compromised pig from the perspective of veterinarians and farmers;
- To elaborate on factors that influence the mental state and that potentially delay euthanizing a compromised pig from the perspective of veterinarians and farmers.

To reach these goals, a cross-sectional survey was created with an exploratory, ex post facto design oriented on design principles in the field [20,21,22,23,24,25,26]. The topics of the survey addressed the demography of participants, experience and (affective) attitudes about pig euthanasia, decision-making and conduct of euthanasia, (reflective) attitudes and feelings about pig euthanasia, and questions specific to the project CARE PIG.

The number of questions totaled 23 for veterinarians, with five additional questions depending on the selected answers, and 21 questions for farmers, with five additional questions depending on the pig category or selected answers. Open questions concerned how decision-making is carried out, what is the most frequent reason for euthanasia of certain pig categories according to their experience (hybrid), what facilitates and challenges euthanasia (hybrid), and what influences mental state if it depends on the situation (hybrid). The survey for veterinarians additionally asked an open question to describe situations in which they are in charge of deciding about euthanasia compared to farmers (hybrid). Hybrid means that the topic was addressed by a closed question as well [22,23]. This design was chosen to combine the exploratory nature of the survey with the aim of validating assumptions, i.e., to avoid predefined responses from anticipating new findings. Due to the amount of data gathered from this survey, this paper excludes the reporting of open responses regarding the decision-making process and training related to the project CARE PIG, which will be discussed in future work.

The content of predefined answers was either based on the research goals outlined above or defined with regard to the results of previous studies [10,13,27]. In particular, the variables of two previous studies [10,13] were translated into German and used to allow a certain comparability in the discussion of the results.

The answer sets for veterinarians and farmers varied slightly, so two surveys were generated and joined for the analysis. The survey was conducted online with the university-hosted platform LimeSurvey^®^ (version 3.23.1+200825, LimeSurvey GmbH, Hamburg, access via Survey3 Stiftung Tierärztliche Hochschule Hannover). A full version of the questionnaire is available online or by request from the corresponding author (see data availability statement).

The participants were informed twice via newsletters from the interest representation of German pig farmers (ISN) and via email distributions by the project partners. However, how the participants were informed about the survey was not followed up to maintain privacy. Moreover, it was not determined whether the participants accessed the survey through the first or second newsletter. To assess eligibility, the participants were asked if they had euthanized a pig before and whether they were responsible for the conduct, the decision, or both aspects of the process.

Analysis of the quantitative survey data was conducted using SAS^®^ statistical software, version 9.4M7 (SAS Institute Inc., Cary, NC, USA). In the first step, the farmer and veterinarian data sets were combined, and missing values were characterized. Subsequently, a data plausibility check was conducted to ensure correct merging of the data.

Given that some questions were answered by a sub-sample only (e.g., veterinarians only), the number of responses (“*n*”) varies during the reporting of the results. Generally, “n” refers to the number of actual respondents, excluding missing values unless otherwise specified. If some participants provided multiple answers in the open text fields, the number of total responses is outlined by “n-responses”. All data used for the analysis are available on demand (see data availability statement).

Given the exploratory nature of the survey, the analysis of survey results relied mainly on descriptive statistical analysis. While tendencies can be seen and described, tests for significance were omitted. In cases in which the results are reported in percentages, the number was rounded to the nearest full number. Related to this, the sum of percentages may differ from 100%.

The analysis of qualitative answers was conducted using the program f4analyse^®^ (f4 analyse, version 3.4.5 from Dr. Dresing and Pehl GmbH). The analysis addressed the open responses concerning influences on the general decision-making process, where respondents could report both facilitating and challenging influences, as well as additional aspects. In the program f4analyse^®^, the responses of each participant were marked and grouped into categories of influences according to their meaning and highest grade of detail. For example, if three methods of euthanasia were considered secure, the response was categorized under “methods” with associated risks, and each was categorized under “method dependency”. These categories were defined based on a recent synthesis of influences on the decision-making process [8], although there was flexibility to define new overall or sub-categories. In case a survey respondent reported an additional aspect that was defined as neither a negative nor a positive influence, it was grouped into the corresponding category but reported as “indefinite” in the survey results.

## 3. Results

### 3.1. Characteristics of the Participants

#### 3.1.1. Response

Overall, 121 participants accessed the online survey (74 farmers and 47 veterinarians). Of these, 12 farmers and 8 veterinarians were excluded because they did not provide any answer or ended without providing demographic information (resulting in 62 farmers and 39 veterinarians). Of these 101 participants, 79 respondents conducted the full online survey (48 farmers and 31 veterinarians).

#### 3.1.2. Demography and Background

This survey includes answers from middle-aged farmers (46–55 years old, 31%, *n* = 61), while half of the responding farmers were between 26 and 45 years old. Most of the veterinarians participating in the survey were between 26 and 35 years old (32%, *n* = 38), and the second-most represented group was over 56 years old (29%). This survey primarily included male participants (73%, *n* = 98), especially male farmers (87%, *n* = 60). For veterinarians, both females (45%) and males (53%, *n* = 38) are represented.

Of the participating farmers (*n* = 62), 58% cared for sows, 89% for fatteners, and 60% for weaning piglets. For sow herds, the most frequently mentioned number of animals in stables was 101–200 (mode: 11%); for fatteners, 2001–5000 (mode: 32%); for weaning piglets, 2001–5000 (mode: 15%). The responses of the veterinarians correspond to the herd size pattern of the farmers: approximately one-third of respondents cared for small sow herds of 201–300 (31%, *n* = 36), and 23% for fatteners, with 751–1000 and 1501–2000 pigs, respectively (*n* = 35). The most frequently mentioned herd size for piglets was between 1500 and 2000 (32%, *n* = 34).

Concerning business ownership, the farmers owned the stable in most cases (87%, *n* = 61) and had worked with pigs for more than five years. The veterinarians were self-employed in more than half of the cases (58%, *n* = 38), and only a few had worked less than five years as a veterinarian (16%, *n* = 37).

#### 3.1.3. Experience and Conduct of Euthanasia

To understand more about the conduct of euthanasia on farms, the farmers were asked how often compromised pigs have to be euthanized on average in a month. Most outlined that euthanasia is required less than once a day (73%, *n* = 56) or approximately once a day (23%). When asked further about the conduct of euthanasia, the majority of the responding farmers were responsible for both the decisions about and the conduct of euthanasia (92%, *n* = 62). When asked about recent euthanasia, 78% of the farmers (*n* = 58) and 76% of the veterinarians (*n* = 37) reported having euthanized a compromised pig in the past two weeks.

When asked about responsibilities, the veterinarians indicated that the farmers primarily euthanized compromised pigs (59%, *n* = 32), while in some cases, both the farmers and the veterinarians were responsible (16%, *n* = 32). The respondents were asked to provide specific cases where veterinarians were responsible rather than farmers. In open response mode, most cases mentioned the conditions of sows (non-ambulatory sows, pregnant sows, and lameness of sows) alongside technical reasons and farmers’ discomfort with specific conditions of the compromised animal.

Apart from responsibilities, the farmers were asked about common methods for the conduct of euthanasia depending on the age category of the pig. The most frequently reported method for sows was electrocution (39%, *n* = 36). For weaning piglets, the respondents outlined the methods of bolt gun stunning/subs. exsanguination (51%, *n* = 37). For fatteners, the respondents also outlined bolt gun stunning/subs. exsanguination in most cases (42%, *n* = 55). For suckling piglets, head strike (and subsequent bleeding) was most frequently mentioned (78%, *n* = 36).

### 3.2. What Are the Common Reasons Mentioned for the Euthansia of Pigs?

To assess what reasons the veterinarians and farmers mentioned for euthanasia, the farmers were asked to provide open responses about why pigs are commonly euthanized depending on the age category of the pig. Subsequently, the farmers and veterinarians were asked to select the three most common reasons for euthanasia out of a predefined list for the respective age category. In cases where specific diseases were mentioned (e.g., *Streptococcus* or CNS infection), the statements outlined suspected etiological causes (abbreviated as “susp”).

#### 3.2.1. Common Reasons Leading to the Euthanasia of Suckling Piglets

The first ranked reason leading to euthanasia of suckling pigs for farmers was substantial loss of weight from the predefined list of responses (50%, *n* = 24) which corresponds to open answers where low viability was reported which includes low birthweight and gain of weight. Veterinarians agreed with the first rank of substantial loss of weight (40%). Apart from the signs of hind-limb weakness and broken bones that were selected of the predefined list from veterinarians only, all reasons of the predefined list were selected. Openly reported by farmers but not represented in the predefined list of reasons are, among others, injuries/trauma, anomaly, and infection.

#### 3.2.2. Common Reasons Leading to the Euthanasia of Weaning Piglets

The first ranked reason leading to the euthanasia of weaning piglets cited by the farmers and veterinarians was clinical signs of non-ambulatory due to suspected CNS infection (35% of farmers, *n* = 23; 37% of veterinarians, *n* = 30). When the farmers were asked openly about reasons, a susp. CNS infection with *Streptococcus suis* was mentioned, along with the disorder known as runting pig syndrome, which describes low-weighted pigs in a poor general condition. Selected by the farmers only was the hernia disorder. Selected by the veterinarians only were signs of hind-limb weakness. Mentioned in open responses but not in the predefined list of reasons for euthanasia were, among others, suspected edema disease, trauma, and organ failure.

#### 3.2.3. Common Reasons Leading to the Euthanasia of Fattening Pigs

The first ranked reason leading to the euthanasia of fattening pigs cited by the farmers was clinical signs of non-ambulatory due to lameness (26%, *n* = 47), which corresponds to the openly reported reasons. The veterinarians ranked non-ambulatory due to lameness, lameness, and tail-root injury equally as reasons for euthanasia (each 23%, *n* = 30). Diarrhea was not selected from the predefined list, although all other options were selected by both groups.

Mentioned by the farmers in their open responses but not in the predefined list were, among others, injury, abscesses, cannibalism, organ failure, bloated belly and runting pigs, and symptoms describing a compromised locomotory tract.

#### 3.2.4. Common Diseases, Disorders, and Clinical Signs of Sows

The first ranked reason leading to the euthanasia of fattening pigs cited by the farmers was clinical signs of lameness (42%, *n* = 24), which corresponds to the openly reported reasons. From the perspective of the veterinarians, clinical signs of non-ambulatory due to lameness (63%, *n* = 30) were mentioned as well. In almost all of the openly reported results, diseases, symptoms, or disorders of the locomotory tract were mentioned. Exceptions in this regard were dystocia, failure of treatment, organ failure, and trauma.

Without anticipating the discussion, the survey results indicate that reasons for euthanizing pigs differ depending on the age of the pig and between veterinarians and farmers in cases of fattening pigs and sows. However, assessment of these differences shows that both groups cited a compromised locomotory tract as a common reason. Veterinarians also reported tail-root injuries, which may result in infection and subsequent lameness. However, this point needs further validation.

A comparison of the openly provided responses and the ranking of the predefined list suggests that assumptions about the most frequent reasons mentioned for euthanasia are supported. The additional reasons reported by the farmers ranged from colloquial or specific clinical signs to diseases and disorders, but also to considerations such as treatment failure. This finding will be discussed further with reference to the level and validity of the reported clinical signs, diseases, and disorders commonly reported for the euthanasia of pigs.

### 3.3. Reported Influences on the Decision-Making Process and Mental State

Once veterinarians or farmers detect a compromised pig based on typical diseases, disorders, or clinical signs, they evaluate whether it is the right time to separate, treat, cull, or euthanize the affected pig. This decision can be challenging and is shaped by several factors. Due to this, the participants were asked openly about influences during the general decision-making process and their situational mental state related to the euthanasia of a compromised pig.

#### 3.3.1. Influences on the General Decision-Making Process

A total of 26 veterinarians and 41 farmers provided open responses about influences on the general decision-making process. Of these, 63 participants reported facilitating factors (38 farmers and 25 veterinarians), 61 reported challenging factors (37 farmers and 24 veterinarians), and 16 described further aspects (9 farmers and 7 veterinarians). The following tables document the commonly mentioned responses (“such as”) and the frequency count of the respective factor (“|x”). To facilitate reading, only the most frequently mentioned categories are outlined in this chapter, while the others are provided in Appendix A.

##### Influences on the Farm

One of the first steps of the decision-making process is to identify and examine the individual pig of concern. Influences related to the category of assessment conditions were mentioned by the participants 10 times. The responses specified the conditions and the timing of assessments. For example, the farmers reported that moving pigs from one pen to another (change of location) facilitates identification, while seasonal businesses like harvesting may be challenging to conduct. Influences on the proper conduct of examinations further embraced having enough light, time, and the chance to conduct the assessment in a calm context. In some cases, animals may have to be presented to veterinarians before decision-making, where a preference for routine visits was outlined compared to additional visits (Appendix A, Table A1).

To evaluate the state of an individual pig, certain clinical signs gained a lot of attention. More precisely, factors describing the category of attributes of clinical signs were mentioned 49 times (Table 1). One attribute was the validity of clinical signs, which refers to the agreement among observers on how to decide about the present and further courses. Hence, the farmers and veterinarians emphasized that agreement with peers on the farm and having a clear diagnosis facilitate decision-making. The relevance of valid clinical signs was also emphasized through the interest in clear guidelines that have been signed by evaluators like public veterinarians.

Another attribute of clinical signs is the ability to grade the condition of a pig and to trace how the condition evolves over time (gradability, Table 1). In this respect, perceiving obvious signs of sickness or signs of a bad general condition was outlined to facilitate the decision to euthanize a compromised pig. Correspondingly, the farmers and veterinarians reported that it is challenging to euthanize a pig who shows the will to live, takes up food and water, or even shows signs of well-being.

Concerning the category of organizational and managerial influences, the survey respondents outlined 19 related aspects (Appendix A, Table A2). The veterinarians emphasized that being informed early about a particular case, and being informed about the farm more generally, facilitates decision-making. Guidelines and protocols, as well as awareness of welfare regulations, were also mentioned as facilitators. The role of human resources, in terms of available and capable personnel, was reported to influence decision-making as well. Another salient topic for the participants was the veterinarian–farmer interaction. Trust, communication (or consultation), and familiarity with one’s veterinarian/farmer were also both indicated to facilitate the decision to euthanize a compromised pig.

For the category of work context, the participants reported 21 factors. Given that the workload is high, the availability of equipment and logistics that afford quick conduct of euthanasia were described as facilitators. The euthanasia methods of electrocution and a CO_2_ box were positively mentioned, in contrast to the method of bolt gun/subs. exsanguination by both the farmers and veterinarians (Appendix A, Table A3).

One last set of influences relates to the category of cultural aspects (11 counts), which addresses, for example, accountability and focus of peers during work on farms. The farmers mentioned tension at work and the need to conduct euthanasia immediately as challenging factors. Overall, the influences in this category suggest that the emphasis is on routines and the herd rather than on the individual handling of compromised pigs. Operational blindness was mentioned by a farmer in this respect as well. Last but not least, a general lack of trust was mentioned as a challenging factor (Appendix A, Table A4).

##### Influences of Individuals

Apart from factors on the farm, influences may relate to the individual human who decides whether or not to euthanize a compromised pig. The survey respondents outlined influences related to the categories of knowledge and skills three times, experience five times, and training once. For example, veterinarians outlined a lack of specific knowledge to identify injuries and diseases as a challenge. In contrast, experience with euthanasia and farming were outlined as facilitators in the decision to euthanize a compromised pig (Appendix A, Table A5)

The most highlighted factor in the category of individual influences, however, was the justification of euthanasia (mentioned 41 times). Among the justifying reasons, the perception of euthanasia as a sign of animal care or as a necessity to alleviate pain and suffering were mentioned as facilitating factors. Seeing euthanasia as a last resort was also mentioned as a positive factor. Similarly, the veterinarians and farmers reported that knowing that the prognosis is infaust facilitates the decision-making process. Correspondingly, doubts about the prognosis or alternate decisions were perceived as challenges in the decision-making process (Table 2).

Concerning the category of attitudes and feelings toward euthanasia (Appendix A, Table A6), 19 influences were mentioned in total by the farmers and veterinarians. One prominent topic was the role of failed hope—for example, in cases where treatment was provided but failed to improve the condition. The farmers reported emotional reasons such as a bond with the animal and personal daily state. In addition to this, discomfort with the conduct of euthanasia and attributes of the method (such as the risk of handling and aesthetics related to exsanguination) were mentioned as influences on the decision-making process. Additionally, many farmers reported an internal conflict and a feeling of wasting food, i.e., a lack of appreciation for the compromised pig as food when the injury or disorder was marginal, yet the whole body of the pig had to be discarded due to euthanasia.

##### External Factors

In addition to influences relating to individual humans, external factors may also shape the decision-making process (mentioned 18 times). The reasons described concerns about public expectations and pressure. Among these, the risk of generating bad publicity and being confronted with pressure from the media, welfare institutes, and official public veterinarians were mentioned (Appendix A, Table A7).

Influences related to the category of economic considerations were mentioned 28 times, representing considerations about the (loss) of profit, costs of conducting euthanasia, or costs of the treatment, which were often outlined as negative influences on the decision-making process by both the farmers and the veterinarians (Table 3).

#### 3.3.2. Mental State and Related Influences

The participants were asked how they rate their state of mind when having to perform euthanasia. More than one-third of the respondents reported having a negative state of mind (35%, *n* = 78), and another third stated that it depends on the situation (31%). The rest of the respondents indicated being neutral (26%) or positive (9%). In comparison, the farmers tended to be more negative (45%, *n* = 47) than the veterinarians (19%, *n* = 31), who tended to be rather neutral (36%) or reported that their state would depend on the situation (36%).

Those respondents (*n* = 26) who reported a negative state of mind were asked if there were one or more specific methods of euthanasia that were specifically distressing. Of these, 27% mentioned bolt gun stunning (subs. exsanguination) and head strike (subs. exsanguination). Meanwhile, 23% outlined a combination of bolt gun stunning (subs. exsanguination), head strike (subs. exsanguination), and bolt gun and subsequent CNS destruction (pithing).

Those respondents who reported that their state of mind depends on the situation were asked to report openly about specific situations to understand more about their thoughts (*n* = 23). In several cases, the respondents stated that euthanasia is generally aversive. While they perceive themselves as rather unemotional, the feeling also depends on their daily state. However, if disagreement about the further course of the pig occurs, the prognosis is unclear, or the overall state of the pig is difficult to assess, the mental state is negatively affected.

The veterinarians outlined that the decision to euthanize an animal is part of their job (being responsible and accountable). Both the veterinarians and the farmers reported that long-time suffering and extended care during the treatment of a pig may lead to grief after euthanasia. In contrast, when euthanizing for a good reason, such as being humane or ending suffering, the respondents perceived a positive mental state.

Without anticipating the discussion, influences affecting the mental state thus appear to correspond to those reported for the general decision-making process.

### 3.4. Attitudes about Euthanasia and Pigs

Certain characteristics of farmers and veterinarians may shape the decision-making process, such as empathy, feelings, emotions, and attitudes about euthanasia. The responses to the related questions (oriented on [10,13]) are summarized below. The sequence of figures and bars corresponds to the variable sequence reported in the text.

#### 3.4.1. Affective Attitudes and Knowledge (Figure 1)

At the beginning of the survey, the participants were asked how much they agreed with statements regarding their responsibility to make a timely decision (know responsibility) and their knowledge that pigs with certain conditions must be euthanized immediately (know immediately) when it comes to the euthanasia of a compromised pig. In general, the level of agreement with the statements was very high in both groups, with the veterinarians agreeing more strongly than the farmers in both cases. One farmer somewhat disagreed with the second statement, and one veterinarian disagreed with both statements.

Apart from questions addressing the perceived knowledge, questions concerning confidence in decision-making were posed. The participants were asked whether they agree that they can evaluate sick or injured pigs (can evaluate state), are confident in making good euthanasia decisions when needed (confidence good decisions), can determine the right time for euthanasia (confidence right time-point), and can conduct euthanasia securely (confidence conduct). The overall agreement was high for each statement, particularly for conducting euthanasia securely.

Concerning the rate of full agreement, however, the response pattern of the farmers deviated from the knowledge-based questions and to the veterinarians. While more than half of the veterinarians fully agreed with being confident making good decisions, only one-third of the farmers agreed. This pattern appeared for the statement concerning confidence to make good euthanasia decisions when needed as well. The lowest agreement from the farmers and veterinarians was related to being confident in determining the right time for euthanasia.

In summary, the respondents generally indicated high agreement with the statements and, thus, a high perceived level of knowledge (and confidence) about the euthanasia of a compromised pig. The lowest agreement was found for statements pertaining to confidence in making decisions about euthanizing a compromised pig. One respondent who rather disagreed appeared to be a young female veterinarian who had been practicing for less than five years. Those farmers who disagreed were 26–55-year-old men who had worked on pig farms for more than five years.

**Figure 1:**
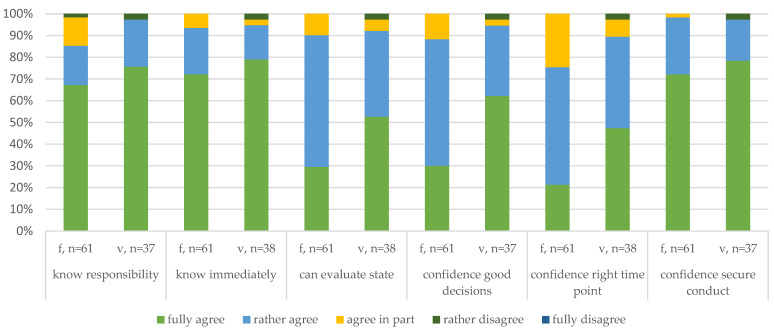
Comparison of the affective responses of the farmers (f) and veterinarians (v).

#### 3.4.2. Reflective Attitudes and Knowledge 

Corresponding to the topics of affective questions, reflective answers were gathered at the end of the survey. Given the relation of these statements to those described in the previous paragraphs, the findings were already interpreted and commented on at that stage, to facilitate the overall discussion in the subsequent chapter.

##### Delay of Decision-Making (Figure 2)

Apart from facilitating or challenging the decision-making process, the participants were asked to rank statements from a list of predefined reasons. The reason that generated the most agreement among the farmers (36%, *n* = 47) and the veterinarians (39%, *n* = 31) was insecurity in defining the chance of healing (chance of healing cannot be foreseen).

For the farmers, these reasons were indeed mentioned as the first and second reasons for the delay in euthanasia. Farmers who ranked the chance of healing first chose, second that the level of suffering cannot be defined (17%, *n* = 46). The farmers who ranked the chance of healing second also ranked, in most cases, insecurity about whether reasonable grounds for euthanasia already exist (15%, *n* = 47). For the veterinarians, their second rank was the misinterpretation of a chance of healing (31%, *n* = 29).

Overall, evaluating the frequencies of the first three ranks—insecurity in defining (and misinterpretation of) the chance of healing, the extent of suffering, and uncertainty if a good enough reason exists—gained the most agreement. This result emphasizes that interest in valid prognosis (security in defining the progress of development over time) is high, which corresponds to the ranking of the justification of euthanasia as a last resort in the open responses.

Another factor that was reported openly was disagreement among evaluators. In the ranking of the reasons for delay, the veterinarians selected this aspect more frequently than the farmers, which also corresponds to previous findings.

Overall, all statements on the provided list were selected as among the first three reasons for delay in euthanasia. Since these statements correspond to openly reported reasons, the findings suggest that factors influencing the general decision-making process contribute to delays in euthanasia. Therefore, the results emphasize the importance of understanding these influences to develop strategies for ensuring timely euthanasia in the future.

**Figure 2:**
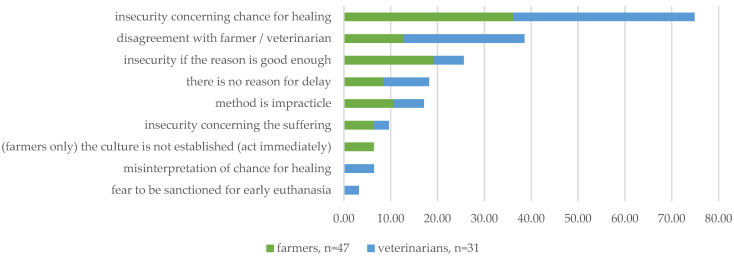
Reasons ranked first for delay in euthanasia (comparison of the frequency of first-ranked reasons between farmers and veterinarians).

##### Decision-Making Behavior (Figure 3 and Figure 4)

At the end of the survey, the participants were asked how much they agreed with several statements related to decision-making behavior. Similar to the affective statement responses, the overall agreement with each statement was high; however, the full scale of responses was used. The majority of the respondents agreed that they had no problem with conducting euthanasia (no problem conduct), but 15% of the respondents disagreed. Compared to the outlined confidence in performing euthanasia beforehand, it seems that agreement declined, and the responses may be more reflective after the survey.

When asked whether it is difficult to decide about euthanasia in certain situations (certain situations difficult), 38% agreed but also disagreed. This finding corresponds to previous responses that describe how the impact of euthanasia is situational; instead of manifesting in one certain situation, the mental state depends on the conduct or attitude of the respondent, which was openly reported beforehand.

Since insecurity in defining the chance of healing was outlined as a challenging factor for timely euthanasia, responses to the statement that it is easy to evaluate the chance of recovery were stunning (easily evaluate recovery). The overall agreement of the participants was very high, and one veterinarian fully disagreed, while two farmers somewhat disagreed. These results suggest that the participants self-reported a high skill or that the terms (evaluating of healing, recovery, or the process of making a prognosis) were understood differently than intended by the participants. Compared to the respondents who disagreed with the affective statements, different veterinarians and farmers disagreed with the current statements.

Another statement related to the decision-making behavior addressed the time taken to evaluate the progress of a pig (tend to fast decisions). More than half of the farmers and veterinarians reported deciding quickly about euthanasia in comparison to others. Since confidence in defining the prognosis, the chance of healing, and determining a good time for euthanasia were outlined as challenging yet important skills for the participants, making fast decisions may be a promising variable for assessing the coping behavior in decision-making processes in future studies.

**Figure 3:**
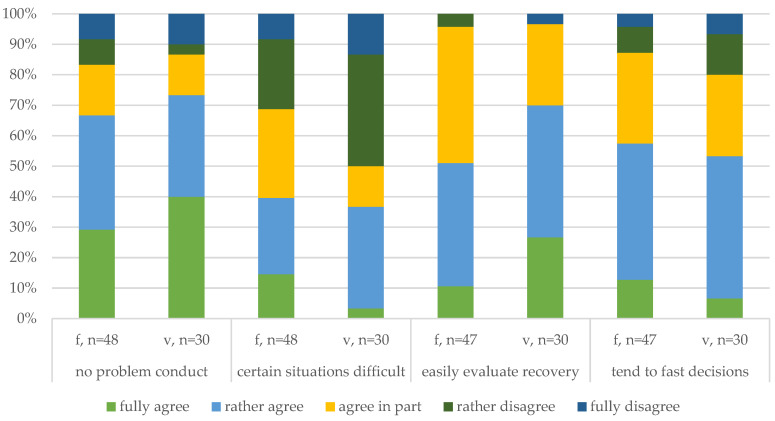
Comparison of the reflective decision-making behavior (1/2) of the farmers (v) and veterinarians (v).

To understand how the participants validate decision-making in difficult cases, the veterinarians were asked about their agreement using the literature instead of talking with colleagues to improve understanding (consultation behavior). More than half of the veterinarians disagreed overall, which suggests that communication with colleagues is one of the dominant modes for improving decision-making related to complicated cases. Instead of asking colleagues, the farmers were asked whether they agreed with consulting their veterinarian to learn more about difficult cases. While most of the participants agreed with consulting their veterinarian, 17% of respondents disagreed. This finding emphasizes the importance of communication (interaction and agreement among veterinarians, farmers, and evaluators). Furthermore, it corresponds to open reports that veterinarians prefer consultation in particular cases and may explain why the ranking of common reasons for euthanasia differed between the veterinarians and farmers.

In addition to consultation with peers, the veterinarians and farmers were asked about participation in further education and training to ensure that their decisions to euthanize a pig are up to date (further training), where overall agreement was reported.

Apart from learning through courses, the role of experience can influence decision-making regarding euthanasia. The respondents were asked if the need to do the right thing increases the more often they euthanize compromised pigs (more experience—right decision). No respondent fully disagreed, and overall agreement was high.

**Figure 4:**
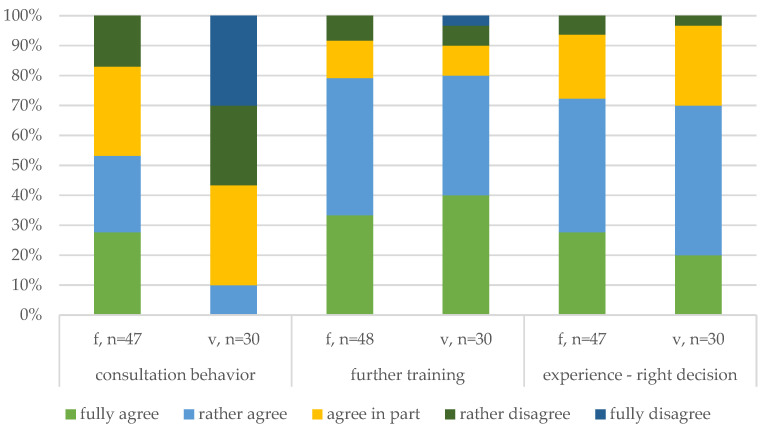
Comparison of the reflective decision-making behavior (2/2) of the farmers (f) and veterinarians (v).

##### Workplace Factors (Figure 5)

To complement the results on perceived knowledge, statements evaluating the role of workplace influences were included. The participants were asked about their agreement with relying on their peers to monitor compromised pigs while they are away from work. Almost half of the veterinarians agreed overall, but the more common response was partial agreement. The farmers showed stronger overall agreement, though 21% either somewhat or fully disagreed. These responses may depend on the availability (and capability) of colleagues and the conditions of the respective workplace, which corresponds to previously reported influences of human workforce factors on the farm.

Related to the assessment of individual pigs on the farm, the participants were surveyed on the role of time for early identification (time—most important factor). Both the farmers (42%) and the veterinarians (50%) reported full agreement, and 83% of all participants agreed overall, compared to 5% disagreement. This finding is noteworthy since only one participant outlined time or timing in their open responses. These results suggest that conditions on the farm and during assessment are very important when caring about compromised pigs and for making timely decisions about the euthanasia of a compromised pig.

**Figure 5:**
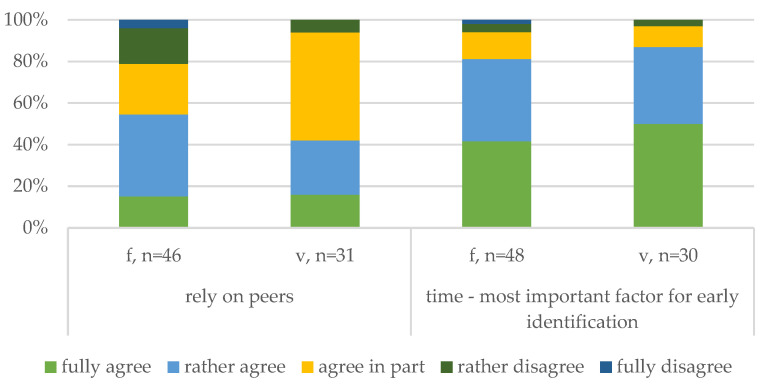
Comparison of workplace factors of the farmers (f) and veterinarians (v).

##### Feelings and Emotions (Figure 6)

The participants were asked about emotional issues, such as feeling bad about conducting euthanasia despite knowing it is the right thing (feel bad despite right decision). Half of the farmers (53%) and approximately 73% of the veterinarians disagreed. Of the 18% of respondents who agreed overall, only a few disagreed with feeling confident in conducting euthanasia or about having no problem. While statistical tests were omitted due to the number of participants in this survey, this statement may be further evaluated to assess emotional involvement in the conduct of euthanasia in future studies.

Empathy in a person may also be associated with emotional involvement, either as a personal affect toward the animal or as a state attributed by the observer to the animal of concern [13] (p. 951). To explore the perspective of German pig farmers and participants on this topic, the participants were asked if imagining how a pig feels is something they do often (imagine feelings). More than 40% of the farmers and veterinarians agreed overall, but one-third of the respondents disagreed. Hence, this statement helps to reveal varied attitudes within the groups. Similar to empathy affect, the distribution of responses concerning empathy attribution was dispersed. The participants were asked whether they agreed that pigs are generally able to feel sadness (pigs feel sadness). A total of 44% of the farmers and 53% of the veterinarians agreed overall, compared to 33% of the farmers and 33% of the veterinarians who disagreed.

Compared to this pattern, the response to the last statement about pigs being sociable animals (pigs are sociable) was more moderate. The veterinarians showed exceptionally strong agreement (90%), while the farmers showed strong agreement (58%) but also expressed disagreement (9%).

In summation, the reflective responses gathered at the end of this survey support and enable a more detailed consideration of influences on the decision-making process. Additionally, the statements related to empathy seem to facilitate the comparison and grouping of responses of both farmers and veterinarians.

**Figure 6:**
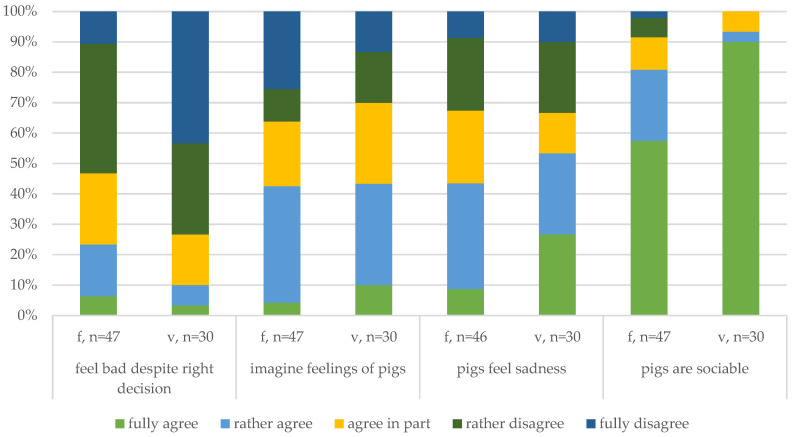
Comparison of the empathy and feelings of the farmers (f) and veterinarians (v).

## 4. Discussion

The decision to euthanize a compromised pig has received considerable attention in recent pig production. For both farmers and veterinarians, it can be challenging to define the right time for euthanasia. This study collected and compared the responses of pig farmers and veterinarians in Germany on this topic. Alignment between the findings and the results of previous studies and whether further implications can be drawn are discussed in this section.

### 4.1. Methodological Considerations

The number of full survey responses was higher than expected cf. [12,13,14], and the abbreviation pattern does not suggest any flaws in the structure of the survey. Self-reported answers need to be interpreted with caution (e.g., due to bias). However, the open answers were evaluated by the author team and with the help of a hybrid design. The results cannot be generalized, as the sample is not representative of the distribution of male and female respondents and length of experience in stables or veterinary practice. Moreover, some analyses could not be stratified, such as the average euthanasia rate per month depending on herd size. Overall, however, this online survey provides a first insight into the perspective of German farmers and veterinarians on the timely euthanasia of a pig.

### 4.2. Diseases, Disorders, and Clinical Signs

One goal of this study was to outline the reasons veterinarians and farmers mention for the euthanasia of certain pig categories. From the perspective of farmers and veterinarians in Germany, the clinical signs of substantial loss of weight for suckling piglets and of being non-ambulatory for weaning piglets and fatteners were ranked as the most important reasons. For sows, the farmers ranked lameness and the veterinarians ranked non-ambulatory as the most important reason for euthanasia. In many cases, the veterinarians and farmers agreed on the ranking of common reasons. Moreover, the experiences reported freely by the participating farmers and veterinarians correspond to those ranked from the predefined list. Additionally, all types of reasons were mentioned and selected (i.e., clinical signs, diseases, and disorders, including suspected etiological diagnosis). These results suggest that an appropriate level of knowledge about common diseases, disorders, and clinical signs is available. Moreover, the commonly reported reasons correspond to previous publications, such as for piglets [3,4,16,28] and sows [4,6,29].

However, a closer look at the type of reported signs shows that the respondents selected clinical signs rather than diseases or disorders first. This finding corresponds to previous studies [4,6,8] and supports the idea of focusing on the level of clinical signs to harmonize the reporting of observations in stables [8,30]. From the perspective of clinical reasoning, harmonizing the level of communication may facilitate the acquisition and sharing of knowledge, which is promising for future training approaches [19,30]. At present, the set of appropriate clinical signs is open to debate. The terms runting pig, low viability, and lameness are vague and need to be specified to allow for the description and comparison of conditions of compromised pigs in more detail [29,31]. In addition, an evaluation of the timeliness of signs is needed since obvious symptoms like cachexia refer to conditions where the right time for euthanasia has been missed from the perspective of the authors. The specificity of certain clinical signs needs to be improved as well, since broken bones and immobility, for example, may both be subsumed by the symptom of a compromised locomotor tract. Some studies have already elaborated on the clinical signs associated with timely euthanasia [2,4,7]. Future studies should increase this evidence and generate a valid taxonomy that can be shared for evaluating the welfare of compromised pigs.

Related to the discussion of clinical signs are the suspected etiological diagnoses being reported by the respondents as well. It is important to know why, in a patho-etiological sense, a pig died or was euthanized in order to learn how to improve diagnoses, treatment, and prognoses in future cases [12]. In this regard, the idea of joining post-mortem examinations with on-farm examination records for analysis is supported [5,6,8,12].

In summary, the results emphasize that more research is needed to improve the taxonomy of reasons and to generate a common language for veterinarians and farmers to elaborate on the conditions of compromised pigs. In particular, improving the description and early identification of pigs with lameness, loss of weight, or non-ambulatory conditions is at stake. The design of long-term studies to follow-up compromised pigs in stables appears to be most promising in this respect.

### 4.3. Influences on the Process

Another goal of this study was to shed light on influences on the decision-making process, the delay of decisions, and the effects on one’s mental state. Overall, the results showed that German participants agree with the importance of timely euthanasia of a compromised pig, but their mental state depends on the situation. Although the respondents did not necessarily feel emotional about the pig of concern, influences during the decision-making process can be demanding.

In summation, the responses from German participants match the synthesis of influences on the decision to euthanize a compromised pig in most respects [8]. Moreover, they resemble the findings in previous studies, where difficulties were reported to result from the nature of euthanasia as both an act and a decision-making process [13]. Hence, the results of this study corroborate previous assumptions about influences on the euthanasia of a compromised pig.

Looking in detail at influences on the decision-making process, the German participants did not elaborate on the role of gender and rarely on the role of required skills compared to other studies [4,27]. Most emphasized was the role of the validity and gradability of clinical signs, the justification of euthanasia as a necessity or last resort, and the role of public pressure or economic considerations.

Firstly, this ranking highlights the importance of research on (valid) clinical signs. The most important reason for delay was the difficulty in evaluating the chance of healing. This is closely connected to determining a clear diagnosis and prognosis for the further course. Hence, the findings of this study suggest that enhanced knowledge about clinical signs would enhance the understanding of the delay of timely euthanasia as well.

From a higher-level perspective, the interest in understanding valid clinical signs and making accurate diagnoses and prognoses reflects a broader interest in enhancing clinical reasoning skills. For training approaches, this perspective can help to define a set of learning goals and modules that go beyond sharing knowledge about the kind of diseases commonly leading to euthanasia. However, one aspect of valid clinical signs that was noted was the agreement with observers. Disagreement, moreover, was ranked highly as a reason for delay from the perspective of veterinarians. On the one hand, the agreement may reflect an interest in security that a certain condition of a pig requires euthanasia. In this case, knowledge about valid clinical signs would facilitate agreement in the long term. On the other hand, the agreement may also reflect an interest in satisfaction with the veterinary–farmer relationship. The fact that trust, the atmosphere on the farm, and familiarity with another were mentioned as influences on the decision-making process also supports this interpretation. Future studies have to assess both assumptions regarding the role of agreement when researching and educating people about the topic of euthanasia of pigs. Previous studies have already elaborated on the role of veterinary client–patient relationships and have suggested that veterinarians may have to train farmers about the topic [12]. In this respect, the findings of this survey suggest that consultation skills or consultation guidelines about euthanasia processes would enhance the handling of the topic on farms in the future.

Coming back to the ranking of influences, the topic of justification of euthanasia needs further attention. This topic is debated not only with regard to compromised pigs but also concerning companion animals, as the elaboration of reasons for euthanasia has recently gained attention [32]. Findings from this survey suggest a re-evaluation of arguments like “euthanasia is necessary” or “a last resort due to an infaust prognosis” is required, with the help of ethical concepts and models, in the future [8,33,34]. For example, it may help differentiate between justifying the decision with the help of medical considerations and explaining the decision related to economic and emotional considerations [32] (p. 15). The results on this topic are likely to complement research on mental health [14], since the participants in this study emphasized good reasons as facilitating and economic considerations as challenging the decision to euthanize a compromised pig.

The role of external and economic factors is another point of discussion. While avoiding unnecessary suffering is the first premise when evaluating the euthanasia of a pig, the economic feasibility of care and public resonance is a major topic for pig farmers and veterinarians. In our findings, the veterinarians reported economic considerations in general, while farmers reported losses as challenges. That economic considerations are important and how they frame the euthanasia decisions of veterinarians has already been shown by in-depth interviews [35]. At present, there is no study in Germany that evaluates how the cost of care and healing compares to the cost of euthanasia when the chance of healing is ambiguous. Previous publications suggest that the results of such a study would corroborate on-farm evaluation plans [16]. However, such a study would surely raise ethical and societal concerns about whether the cost of care should matter when deciding on the timely euthanasia of a pig. The acceptance of hospital pens has been shown to be difficult in international studies [36], and only a few guidelines about further care have been provided by public officials in Germany [37]. Hence, to address this challenging topic, a global understanding that prolonged care of a compromised pig may include accepting a few days of suffering, a point that public veterinarians need to consider during controls as well, is needed.

One way to provide support for farmers and veterinarians (to grant time for healing) is to generate further evidence about the course of diseases in a compromised pig. In addition to the discussion about clinical signs above, research on the typical clinical signs under treatment regimes in stables should lead the focus.

A way to generate global understanding, however, is to discuss ethical questions such as whether a particular certainty (or chance) is enough of a reason to decide upon the life of an individual pig. Hence, it is a matter of societal debate to define whether knowledge of statistical certainty is appropriate to make a euthanasia decision, while it is a matter of research to elaborate on which statistical certainty can be achieved with the help of valid clinical signs [28].

### 4.4. Attitudes, Knowledge, and Personality

The last and final goal of this study was to assess the specific characteristics relevant to research on the topic of euthanasia of a compromised pig. In particular, the stance of German participants concerning decision-making behavior and personal traits, such as empathy and attitudes toward euthanasia, was assessed.

Similar to the results published in previous studies, the perceived knowledge of euthanasia of the respondents was high [10]. Moreover, the affective and reflective responses generally corresponded with the assumptions discussed in the previous chapters. For example, the participants have difficulties with certain situations but not with euthanasia in general. Based on our results, the most challenging factor in the “attitudes” category for the farmers was emotional strain due to a perceived waste of the animal’s life as food. Previous studies have found that conflict of personal motives or roles can result in mental stress and coping strategies [12,38]. While this survey outlined the facilitating and challenging nature of influences, future studies should assess how to avoid moral distress and compassion fatigue when internal conflict arises.

Our study explored how German survey respondents agree with statements of empathy that have been used in previous studies [11]. The responses deviated between farmers and veterinarians and may be fruitful for future cluster analysis.

The ranking of influences on the decision-making process and delay in euthanasia in our results further emphasized elaboration on personality traits in relation to euthanasia remains a promising research field [14,39]. Apart from empathy, accountability and a sense of responsibility were indicated in our results, yet factors describing personality traits in this study were vague. As elaborated in previous studies [38], multiple responsibilities can be perceived by veterinarians in euthanasia situations, such as their role as advisors, decision-makers, educators, and surveillants. (Self-)perceived responsibilities and roles may frame euthanasia decisions and how veterinarians and farmers interact [39]. In this regard, knowledge of personality traits, roles, or frames may be useful in relation to the topic of improved consultancy and training between veterinarians and farmers [8,12,39,40].

## 5. Conclusions

This article presented the results of a survey about the reasons, influences, and attitudes of German pig farmers and veterinarians regarding the euthanasia of a compromised pig. The results of the online survey allowed us to generate a list of common diseases, disorders, and clinical signs for suckling, weaning, and fattening pigs and breeding sows. Moreover, the results showed that a multitude of influences, due to the work context on farms and economic and personal considerations, shape the decision-making process.

The results often corresponded to those published in similar studies. However, the main influences were the validity of clinical signs, insecurity in defining further progress (and, in this respect, to justify euthanasia), and the role of economic considerations. Implications for future studies were specified in the discussion, and addressed, among other factors, was the need to enhance the understanding of the trajectory of typical clinical signs during treatment, the value of ethical concepts to enhance the understanding of the justification of euthanasia decisions, and—last but not least—the benefit of consultancy models for farmers and veterinarians about euthanasia decisions.

## Figures and Tables

**Table 1:** Attributes of the clinical signs reported by 15 farmers and 12 veterinarians (49 counts in total).

Category	Description	Farmers	Veterinarians
Validity |29	Disagreement among evaluators |11	Facilitating|2, such as agreement with family members, peers, and veterinarians Challenging|1	Facilitating|3, such as agreement with farmers Challenging|5, such as disagreement with farmers
	Disagreement about the case |13	Facilitating|4, such as clear diagnosis, case, and disease Challenging|5, such as unclear diagnosis, case, or disease Indefinite|1	Facilitating|1 Challenging|2, such as unclear diagnosis
	Agreed upon instructions |5	Challenging|1, such as lack of clear instructions	Facilitating|3, such as pictures, guidelines signed by a public veterinarian; at least one treatment should always be granted Challenging|1
Gradability |20	Body condition score |1		Facilitating|1, such as cachexia
	Obvious signs |7	Facilitating|4, such as obvious or severe signs Challenging|2, such as obvious or minor impairment	Facilitating|1
	Overall state |12 counts	Facilitating|3, such as bad mobility, bad general condition, and apathy Challenging|5, such as good overall condition, will to live, and signs of well-being	Facilitating|2, such as bad overall state Challenging|2, such as good overall condition and interest in feed and water

**Table 2:** Reasons to justify euthanasia reported by 17 farmers and 10 veterinarians (41 counts in total).

Category	Description	Farmer	Veterinarian
Necessity |18	Necessary to stop suffering |9	Facilitating|7, such as understand necessity and stop suffering Challenging|1	Challenging|1, such as (mis-) understanding animal protection
	Decision serves pig welfare and stops pain |9	Facilitating|5, such as avoid pain and serve welfare	Facilitating|2, such as avoid pain Challenging|1 Indefinite|1
Last resort |23	No other feasible option |23	Facilitating|3, such as treatment not feasible, infaust prognosis, and no chance of healing Challenging|5, such as unclear prognosis Indefinite|1	Facilitating|9, such as infaust prognosis and treatment makes no sense, has no effect, or has no chance Challenging|4, such as unclear prognosis Indefinite|1

**Table 3:** Economic considerations reported by 10 farmers and 9 veterinarians (28 counts in total).

Category	Description	Farmer	Veterinarian
Economic considerations |28	Procedural costs |5	Challenging|2, such as costs of disposal Indefinite|1	Challenging|2, such as costs of treatment, and costs for euthanasia
	Profit calculation |13	Facilitating|1, such as “small” animal—low losses Challenging|8, such as “good” animal and non marketable animals	Facilitating|1, such as non-marketable animals Challenging|3, such as potentially marketable animals
	General economic situation |10	Challenging|2, such as economic considerations	Facilitating|1, such as economically not making sense not to euthanize Challenging|6, such as economic situation or crisis of farmers Indefinite|1

## Data Availability

The online survey can be accessed online: https://www.tiho-hannover.de/fileadmin/23_Biometrie/Dateien/Zusatzmaterial/Website_OnlineSurveyMaterial.pdf. The data were collected online on an individual basis from farmers and veterinary practitioners. Each participant provided consent with the understanding that data would not be transferred to a third party. Therefore, any data transfer to interested persons is not allowed without an additional formal contract. Data are available to qualified researchers who sign a contract with the University of Veterinary Medicine Hannover. This contract will include guarantees of the obligation to maintain data confidentiality in accordance with the provisions of the German data protection law. Currently, there is no data access committee or another body who could be contacted for the data. However, for this purpose, a committee will be founded. This future committee will consist of the authors as well as members of the University of Veterinary Medicine Hannover and members of the funding institution. Interested cooperative partners who are able to sign a contract as described above may contact the corresponding author.

## Appendix A

**Table A1:** Assessment conditions on the farm reported by 2 veterinarians and 4 farmers (10 counts in total).

Category	Sub-Category	Description	Farmer	Veterinarian
Timing |3	Pig—oriented |1	Incidents related to the caring for pigs	Facilitating|1	
	Business—oriented |1	Incidents related to work or personal concerns	Challenging|1	
	Indefinite |1	Timing is important|1		
Proper conduct |7	Conditions to identify |4	Look closely, organise follow up of animals |4	Facilitating|1 Challenging|1	Facilitating|1 Challenging|1
	Organise the monitoring |3	Pick up/get animal up to inspect |3	Indefinite|1	Facilitating|1 Challenging|1

**Table A2:** Organizational and managerial influences reported by 6 veterinarians and 5 farmers (19 counts in total).

Category	Description	Farmer	Veterinarian
Documentation |2	Records about animal |2		Facilitating|2, such as early information
Protocols |3	Work regulations |3	Challenging|1, such as lack of	Facilitating|2, such as welfare regulation and guideline
Human resources |3	Available personnel |2	Facilitating|1 Challenging|1, such as lack of personnell	
	Capable (authorized) personnel |1	Indefinite|1	
Veterinarian—farmer interaction |11	Trustful interaction |11	Facilitating|2, such as consultation with or instruction by veterinarian	Facilitating|7, such as: trust, communication, cooperation and familiarity Indefinite|2, such as be a consultant

**Table A3:** Work context factors reported by 3 veterinarians and 10 farmers (21 counts in total).

Category	Description	Farmer	Veterinarian
Work load |3	Working environment |3	Challenging|3, such as high workload	
Logistics |4	Sufficiency of the location |4 counts	Facilitating|3, such as quick technique, short ways	Facilitating|1
Technical aspects |14	Maintenance of technique |4 counts	Facilitating|4, such as good techniques or availability of technique	
	Method dependency |10 counts	Facilitating|4, such as CO_2_ box or electrocution Challenging|1	Facilitating|4, such as CO_2_ box electrocution and injection Challenging|1

**Table A4:** Cultural aspects reported by 4 veterinarians and 6 farmers (11 counts in total).

Category	Description	Farmer	Veterinarian
Accountability |4	Stance towards practices |1		Indefinite|1
	Sense of discipline |3		Challenging|2, such a feeling to be responsible Indefinite|1
Orientation |1	Person oriented |1	Challenging|1	
Focus of actions |4	Herd health first |1	Challenging|1	
	Routine work first |3	Facilitating|1 Challenging|1 Indefinite|1	
Operational blindness |1	Always been like this |1	Challenging|1	
Trust |1	Trust in general |1	Challenging|1, such as lack of trust	

**Table A5:** Gender, knowledge, experience and training factors reported by 3 veterinarians and 4 farmers (9 counts in total).

Category	Subcategory	Farmer	Veterinarian
Knowledge and skills |3	General knowledge set |1	Challenging|1	
	Skillset to evaluate |1	Challenging|1	
	Skillset to diagnose |1		Facilitating|1
Experience |5	Repetition of euthanasia conduct |4	Facilitating|1	Facilitating|2, such as routine and experience Challenging|1
	Experience |1	Facilitating|1	
Training |1	Available training |1	Challenging|1, such as lack of training	

**Table A6:** Attitudes and feelings reported by 3 veterinarians and 18 farmers (23 counts in total).

Category	Description	Farmer	Veterinarian
Failure |5	Fail to care enough |5	Facilitating|1 Challenging|3, such as failed hope that treatment works	Challenging|1
Willingness |1	Lack of willingness to perform |1	Challenging|1	
Personality |4	Empathy—caring nature |1	Challenging|1	
	Personality in general |3	Facilitating|1 Challenging|2, such as daily emotional state	
Discomfort/conduct |3	Discomfort with act of killing |2	Challenging|2, such as natural threshold and killing is never nice	
	Discomfort with animal type |1		Challenging|1, such as decide between sow and piglet
Discomfort to method |6	Risk doing the method |2	Facilitating|1	Facilitating|1, such as:method is without risk
	Aesthetics of method |4	Facilitating|2, such as method is without blood and clean Challenging|2, such as bloody methods	
Mental strain |4	Mental—emotional strain |4		

**Table A7:** External factors reported by 4 veterinarians and 8 farmers (18 counts in total).

Category	Description	Farmer	Veterinarian
External factors |18	Public opinion about compromised pigs and euthanasia |7	Facilitating|2, such as avoid bad publicity and pressure of society Challenging|2, such as bad publicity and pressure from society Indefinite|1	Facilitating|1 Indefinite|1
	Public concerns about welfare |2	Facilitating|1	Facilitating|1
	Risk of being seen or convicted |9	Facilitating|3, such as avoid risk being convicted Challenging|3, such as avoid trouble, the right time point and high penalties	Facilitating|1 Challenging|1 Indefinite|1
